# Protein Surface Softness Is the Origin of Enzyme Cold-Adaptation of Trypsin

**DOI:** 10.1371/journal.pcbi.1003813

**Published:** 2014-08-28

**Authors:** Geir Villy Isaksen, Johan Åqvist, Bjørn Olav Brandsdal

**Affiliations:** 1 The Norwegian Structural Biology Center and the Center for Theoretical Computational Chemistry, Department of Chemistry, Faculty of Science and Technology, University of Tromsø, Tromsø, Norway; 2 Department of Cell and Molecular Biology, Biomedical Center, Uppsala University, Uppsala, Sweden; University of Maryland, United States of America

## Abstract

Life has effectively colonized most of our planet and extremophilic organisms require specialized enzymes to survive under harsh conditions. Cold-loving organisms (psychrophiles) express heat-labile enzymes that possess a high specific activity and catalytic efficiency at low temperatures. A remarkable universal characteristic of cold-active enzymes is that they show a reduction both in activation enthalpy and entropy, compared to mesophilic orthologs, which makes their reaction rates less sensitive to falling temperature. Despite significant efforts since the early 1970s, the important question of the origin of this effect still largely remains unanswered. Here we use cold- and warm-active trypsins as model systems to investigate the temperature dependence of the reaction rates with extensive molecular dynamics free energy simulations. The calculations quantitatively reproduce the catalytic rates of the two enzymes and further yield high-precision Arrhenius plots, which show the characteristic trends in activation enthalpy and entropy. Detailed structural analysis indicates that the relationship between these parameters and the 3D structure is reflected by significantly different internal protein energy changes during the reaction. The origin of this effect is not localized to the active site, but is found in the outer regions of the protein, where the cold-active enzyme has a higher degree of softness. Several structural mechanisms for softening the protein surface are identified, together with key mutations responsible for this effect. Our simulations further show that single point-mutations can significantly affect the thermodynamic activation parameters, indicating how these can be optimized by evolution.

## Author Summary

Cold-adapted organisms require specialized enzymes to maintain functional integrity at low temperatures, and psychrophiles express heat-labile enzymes that possess a high specific activity and catalytic efficiency at low temperatures. The high catalytic rates are achieved by enzyme adaptations yielding lower activation enthalpies and entropies than for mesophilic homologs, thereby solving the problem of the exponential rate decrease with falling temperature. However, the structural mechanisms behind this universal property of cold-adapted enzymes remain unknown. By extensive computer simulations, which reproduce both the experimental reaction rates and the characteristic temperature dependence of activation free energies, we show that it is the softness of the protein-water surface that regulates the activation enthalpy-entropy balance. Structural mechanisms behind this phenomenon are identified and our simulations show that single mutations can significantly affect the thermodynamic activation parameters, indicating how these can be optimized by evolution.

## Introduction

One of the most intriguing problems in biology regards the molecular mechanisms involved in adaptive capabilities for life in extreme environments. Cold-adapted organisms have an extraordinary ability to grow in and colonize environments where the temperature is close to the freezing point of water. From the viewpoint of chemical kinetics, a key problem with lowering the temperature is that the enthalpy of activation gives rise to an exponential decrease in enzyme reaction rates according to transition state theory
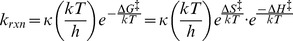
(1)Here, *k_rxn_* is the reaction rate and T the temperature, *κ* is a transmission coefficient, *k* and *h* are Boltzmann's and Planck's constants, respectively, and Δ*G* ^‡^ is the free energy of activation. The latter quantity can be decomposed into entropic (−*T*Δ*S* ^‡^) and enthalpic (Δ*H* ^‡^) contributions and decreasing the temperature from 37°C to 0°C typically results in a 20–250 fold reduction of the activity of a mesophilic enzyme [1]. Survival at low temperatures thus requires that the enzyme kinetics can be adapted to avoid this problem and also that protein stability is maintained in a cold environment.

As a strategy to cope with the strong temperature dependence of the reaction rates, psychrophiles synthesize heat-labile enzymes possessing a high specific activity and catalytic efficiency at low temperatures [2], [3], [4]. It is thus well established that cold-adapted enzymes generally have reduced thermal stability compared to mesophilic orthologues, presumably to counteract the increase in structural rigidity at lower temperatures [1], [5], [6]. However, the change in structural stability does not seem to follow any general rule, but is rather a combination of several factors [5]. More remarkable, however, is the seemingly universal characteristic that catalyzed reactions of cold-adapted enzymes have a *lower enthalpy and a more negative entropy of activation* than their mesophilic and thermophilic counterparts [1], [5], [6]. Overall *activation free energies*, on the other hand, are usually similar around room temperature [5]. The lower activation enthalpy thus makes the rate less temperature dependent (equation (1)) and is believed to be the primary adaption in psychrophilic enzymes [2], [7], [8]. It has long been proposed that cold-adaptation originates from increased flexibility of the active site [6], which could hypothetically yield lower activation enthalpies at the expense of requiring more ordering of substrates and the active site, as the reaction barrier is surmounted (i.e., a more negative Δ*S* ^‡^). However, there seems to be no strong experimental support for this hypothesis and, e.g., X-ray analysis of cold- and warm-active trypsin did not indicate any overall flexibility differences between the two enzymes [9]. Moreover, recent computer simulations of differently adapted citrate synthases showed that the flexibility of the highly conserved active site residues was virtually identical. Instead it was found that differences in protein stiffness outside of the active site appear to be correlated with differences in thermodynamic activation parameters [10].

The origin of catalytic rate optimization in cold-adapted enzymes, in terms of actual structure-function relationships, thus remains rather obscure. Understanding such relationships would not only provide information regarding the evolutionary adaption processes, but potentially also enable rational design of enzymes adapted to low temperature. Computer simulations could provide a unique way of analyzing the reaction energetics of differently adapted enzyme orthologs. However, in order for such a strategy to be viable several criteria must be met. First, analysis of indirect or circumstantial factors (flexibility, electrostatics, hydrophobicity etc.) alone does not suffice for obtaining conclusive evidence. Instead reliable free energy profiles along the reaction pathway must be obtained with high precision. Second, the crucial activation enthalpy-entropy balance for different enzymes must be reproduced by the simulations and the only way to do this is to computationally obtain Arrhenius plots for the activation free energy versus temperature. This involves calculating a large number of free energy profiles at different temperatures so that activation enthalpies and entropies can also be extracted with high precision. Clearly, such extensive sampling by molecular dynamics (MD) simulations precludes the use of most standard QM/MM approaches, but the empirical valence bond (EVB) model [11], [12]. provides a very efficient method for this purpose. Third, provided that the experimentally observed activation enthalpy-entropy balances are captured by the simulations, it must be possible to decompose these into their underlying energy components and ultimately translate them into differences between the enzyme 3D structures and fluctuations.

Here, we report extensive MD/EVB free energy simulations that yield high precision Arrhenius plots for the reactions of psychrophilic and mesophilic trypsins. The calculations reproduce both experimental rates at room temperature and the characteristic relationships between activation enthalpy and entropy for the orthologous salmon and bovine enzymes. The relationship between these parameters and the 3D enzyme structures is reflected by significantly different internal protein energy changes during the reaction. This effect originates from outside of the active site where the cold-adapted salmon enzyme has a higher degree of softness, which is evident from the corresponding potential energy term. We also identify key residues for which simulations predict significantly altered thermodynamic activation parameters upon mutation.

## Methods

Atomic coordinates for psycrophilic and mesophilic trypsin were obtained from the crystallographic structures with PDB entries 1BZX [13] and 3BTK [14], respectively. All EVB calculations were performed with the molecular dynamics package Q [15] using the OPLS2005 all-atoms force field [16], [17]. Additional simulations details are given in Text S1. The EVB reaction surface was calibrated using the imidazole catalyzed methanolysis of formamide in water [18] as a reference reaction (Text S1). The EVB free energy profiles were calculated using the free energy perturbation (FEP) umbrella sampling approach described elsewhere [11], [12]. Each enzyme and water reaction free energy profile involved 500 ps of MD simulation and compromised 51 discrete FEP steps. Thermodynamic activation parameters were obtained from Arrhenius plots based on simulations at eight different temperatures (275–310 K). At each temperature point 100 and 150 independent FEP simulations were carried out, resulting in a total simulation time of 408 and 612 ns for salmon and bovine trypsin, respectively. In addition 100 ns simulation time was performed at the reactant and transition state at 300 K for both systems. Enzyme mutations were created using the builder tool in Mastro 9.1 (Schrödinger, LLC, New York, NY, 2011). The mutated residues were relaxed prior to MD simulation with the clean up geometry tool in Maestro. In order to obtain reliable sampling, the simulations were repeated 20–60 times at each temperature (275–310) for the mutated model systems.

## Results

Serine proteases are enzymes that catalyze the cleavage of peptide bonds in proteins and peptides and have numerous important physiological functions. They have been extensively studied for many decades and the reaction scheme involves formation of a Michaelis-Menten complex, nucleophilic attack by the characteristic serine residue to form an acyl-enzyme intermediate and subsequent hydrolysis of this intermediate to yield the final products [19], [20]. These enzymes have an invariant catalytic triad, which in trypsin is formed by Ser195, His57 and Asp102. The histidine residue acts as a general base for activating the serine side-chain, while Asp102 is essential for stabilizing the resulting protonated form of the histidine [21]. The rate-limiting step of the reaction is generally considered to be the formation of a transient tetrahedral intermediate, the breakdown of which leads to acylated enzyme. The large rate acceleration compared to uncatalyzed peptide bond hydrolysis is primarily accomplished by facilitating formation of the reactive nucleophile and by transition state stabilization. Here, the so-called oxyanion hole, formed by the backbone NH groups of Gly193 and Ser195, also plays a key role by stabilizing the developing negative charge (oxyanion) of the tetrahedral intermediate [21].

### Computational Arrhenius plots show the characteristics of cold-adaptation

**Figure 1:**
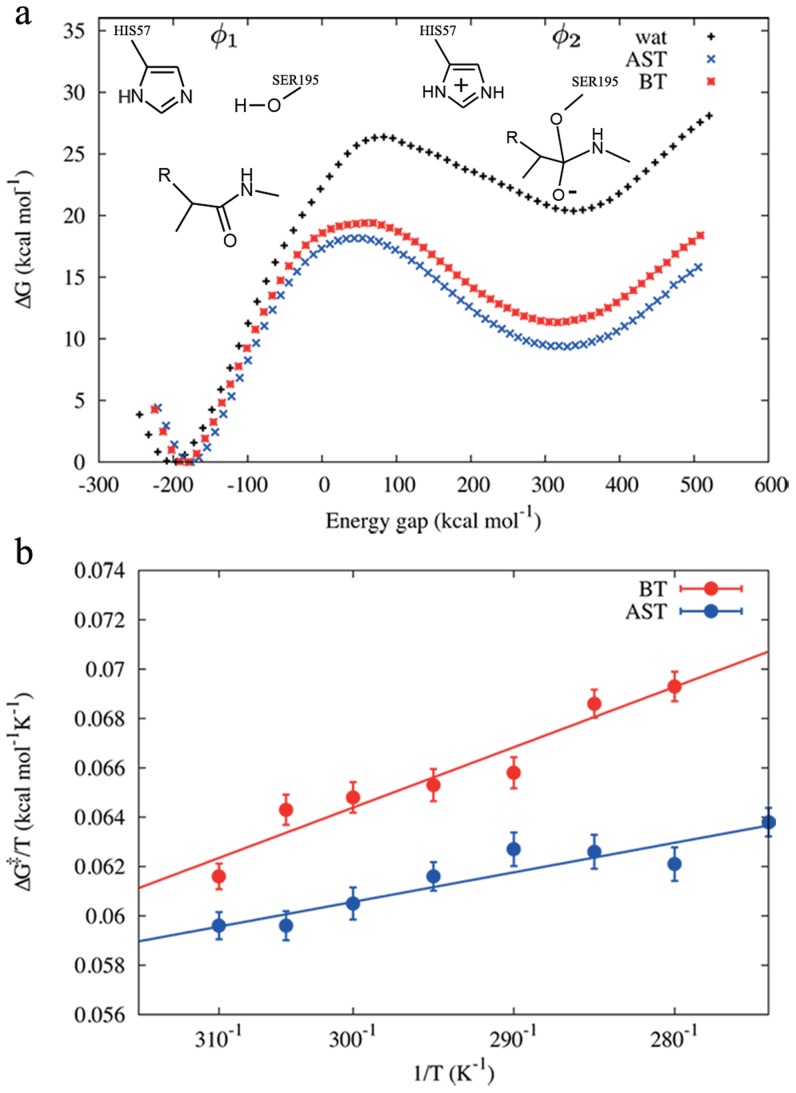
Comparison of free energy profiles and Arrhenius plots. (a) Calculated free energy profiles (300 K) for tetrahedral intermediate formation in the acylation step of bovine trypsin (BT), anionic salmon trypsin (AST) and in the imidazole catalyzed reference reaction in water. (b) The clear difference in slopes from the calculated Arrhenius plots (ΔG^‡^/T vs 1/T) for BT (red) and AST (blue) demonstrate that cold adapted trypsin has a lower activation enthalpy compared to the warm-active ortholog.

We used the reactions of the mesophilic bovine trypsin (BT) and the psychrophilic anionic salmon trypsin (AST) as models to examine the temperature dependence of reaction rates for differently temperature adapted enzymes. The energetics of the rate-limiting formation of the tetrahedral intermediate, using a Cys-Lys-Ala tripeptide as substrate, was calculated by the MD/EVB approach [11], [12]. The results from these simulations at 300 K are shown in Fig. 1a as free energy profiles along the reaction coordinate for the two enzymes. The corresponding free energy profile for the reference reaction used to calibrate the EVB potential (see Text S1), i.e., imidazole catalyzed formation of the tetrahedral intermediate in water [18], is also shown. In order to attain a sufficiently high precision the calculations were averaged over up to 150 independent runs at each temperature (see below). The calculated activation energies at 300 K of 18.2±0.2 kcal/mol and 19.0±0.2 kcal/mol for AST and BT, respectively, are in excellent agreement with the substrate dependent barrier of 15–20 kcal/mol [19]. This difference in activation free energies translates into a 4-fold increase in k_cat_ for AST when compared to BT, which is in remarkable good agreement with experiments that shows 2- to 4-fold increase depending on the temperature [22]. The simulations also clearly demonstrate the large catalytic effect on the reaction for both enzymes. Compared to the uncatalyzed hydrolysis reaction in water, the transition state is found to be stabilized by over 13 kcal/mol [18], [23]. With respect to the imidazole catalyzed reference reaction in solution the corresponding stabilization is about 7 kcal/mol [18].

Since the catalytic rates of the two trypsins at room temperature are well reproduced by the MD/EVB simulations, we can now turn to examine their temperature dependence. Eight different temperatures were chosen in the range of 275 to 310 K and 100–150 independent free energy profile calculations were carried out at each temperature to obtain high precision Arrhenius plots. Activation entropies and enthalpies were then extracted by linear regression from plots of ΔG^‡^/T *vs.* 1/T. The temperature dependence of the activation free energies is shown in Fig. 1b and it can immediately be seen that the psychrophilic enzyme (AST) has a significantly smaller slope than the mesophilic counterpart (BT). The calculated activation parameters for BT are ΔH^‡^ = 20.4 kcal/mol and ΔS^‡^ = 3.5 e.u, while the corresponding values for AST are ΔH^‡^ = 9.9 kcal/mol and ΔS^‡^ = −27.5 e.u. This is thus a remarkable example of enthalpy-entropy compensation where the large differences in ΔH^‡^ are balanced by -TΔS^‡^ contributions at 300 K of −1.4 and +8.3 kcal/mol for BT and AST, respectively (Table 1), to yield similar activation free energies. It should be noted that an increase in the activation free energy of 1 kcal/mol directly translates into a 5-fold decrease in k_cat_. The fact that both the absolute rates at 300 K and the characteristic balance between activation enthalpy and entropy for the mesophilic and psychrophilic enzymes are reproduced by the computer simulations is also remarkable and raises the question of what the structural origin of this effect really is.

**Table 1:** Calculated thermodynamic activation parameters (kcal/mol) for native and mutant bovine (BT) and salmon (AST) trypsins at 300 K.

Enzyme	ΔG^‡^	ΔH^‡^	TΔS^‡^	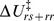 *	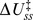 *
BT_native_	19.0±1.4	20.4±1.0	1.4±1.0	14.6±0.7	5.8±1.3
BT_N97Y_	18.4±1.3	10.6±0.9	−7.8±1.0	15.9±1.1	−5.3±1.5
BT_S150D_	18.6±1.3	14.5±0.9	−4.1±1.0	14.9±1.1	−0.4±1.5
AST_native_	18.2±0.8	9.9±0.6	−8.3±0.6	13.1±0.9	−3.2±1.1
AST_Y97N_	18.4±1.1	12.0±0.8	−6.4±0.8	13.9±1.2	−1.9±1.4
AST_D150S_	18.0±1.1	14.7±0.8	−3.2±0.8	11.5±1.9	3.2±1.2

*Subscripts rr, rs and ss denote, respectively, interactions among atoms in the EVB region, their interactions with the surroundings, and the internal interactions within the surroundings.

Error bars denote standard error of the mean.

### Differences in activation parameters are associated with protein stiffness

As far as energetics is concerned it is relatively straightforward to identify the source of the difference in activation enthalpy between the two enzymes. Since Δ*H* ^‡^ = Δ*U* ^‡^+*p*Δ*V* ^‡^, and the pressure-volume term is completely negligible, the activation enthalpy is determined by the corresponding change in internal (total) energy of the system. The latter can be decomposed into contributions from the reacting fragments (i.e., the EVB atoms whose interaction parameters change along the reaction), their interactions with the surrounding protein and solvent, and the interactions within the surrounding environment
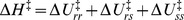
(2)Here, the subscripts *r* and *s* denote the reacting fragments and surroundings (the protein and solvent included in the simulations), respectively. The last term of equation (2) involves very large energies, since it pertains to a huge number of interactions within the surrounding protein and solvent, making it practically impossible to obtain a converged value for this quantity directly from the MD simulations. However, since both Δ*H* ^‡^ and 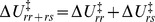 can be evaluated from the trajectories with sufficiently high precision we can still get an accurate estimate of all the terms in equation (2). Table 1 shows this breakdown of the energetics which immediately reveals that the source of the decreased activation enthalpy in the cold-adapted enzyme is not associated with a more favorable 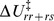 term. Instead it is a significantly lower value of 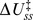 that is responsible for the decrease in Δ*H* ^‡^. Hence, while the internal energy change involving the reacting groups is similar, the contribution from the surroundings is predicted to be about 9 kcal/mol more favorable for AST than BT. It would be desirable to further decompose 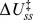 into protein-protein, protein-water and water-water interaction contributions according to
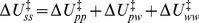
(3)but, again, the energies involved are too large to allow converged direct calculations of these averages. However, from the viewpoint of locality it is reasonable to expect that the two first terms involving protein interactions dominate the reduction in 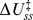 or the cold-adapted enzyme. That is, the protein interactions are likely to respond more strongly to the energy change in the active site, associated with climbing the activation barrier, since the active site is primarily embedded in the protein, which in turn is surrounded by water.

At any rate, we can conclude that the reduction of activation enthalpy in the cold-adapted enzyme originates from interactions outside of the active site. This is perhaps not so strange since all residues surrounding the substrate are conserved between the two proteins, making it more likely that energetic differences are to be found farther away. The fact that the energy cost reflected by the 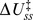 term is lower in the cold-active than the warm-active trypsin further suggests that the surroundings of the active site are effectively softer in the salmon enzyme. In this respect, the term “softness” can be more precisely defined than protein flexibility in general, as it refers to the change in potential energy of the surroundings of the active site as the system moves along the reaction coordinate form reactants to transition state. This potential energy change can thus be viewed as reflecting an effective force constant of the surroundings, which is stiffer in the warm-active enzyme and softer in the cold-active. This brings us back to the possible role of protein flexibility in cold-adaptation.

### Differences in protein flexibility are found on the protein surface

Cold-adapted enzymes are often assumed to benefit from higher flexibility to deal with the decrease in chemical rates and altered structural rigidity at low temperatures. Since the activation entropy is also more negative than for mesophilic homologs, this could be interpreted in terms of an increased flexibility of the active site in the reactant state [8]. This proposal was, however, not supported by Bjelic *et al.* who evaluated the positional root-mean-square fluctuations (RMSF) of the key residues in the active site of different temperature-adapted citrate synthases [10]. They demonstrated that the active site and substrate mobilities were virtually identical and found no indication of the cold-adapted enzyme having larger active site RMSFs compared to the heat-adapted enzymes. The fluctuations obtained with a spherical boundary model were also found to be virtually identical to those obtained with a much larger simulation system simulated using periodic boundary conditions. It should be noted that the present calculations were carried out with the entire protein immersed in a spherical droplet of water (Fig. S1).

**Figure 2:**
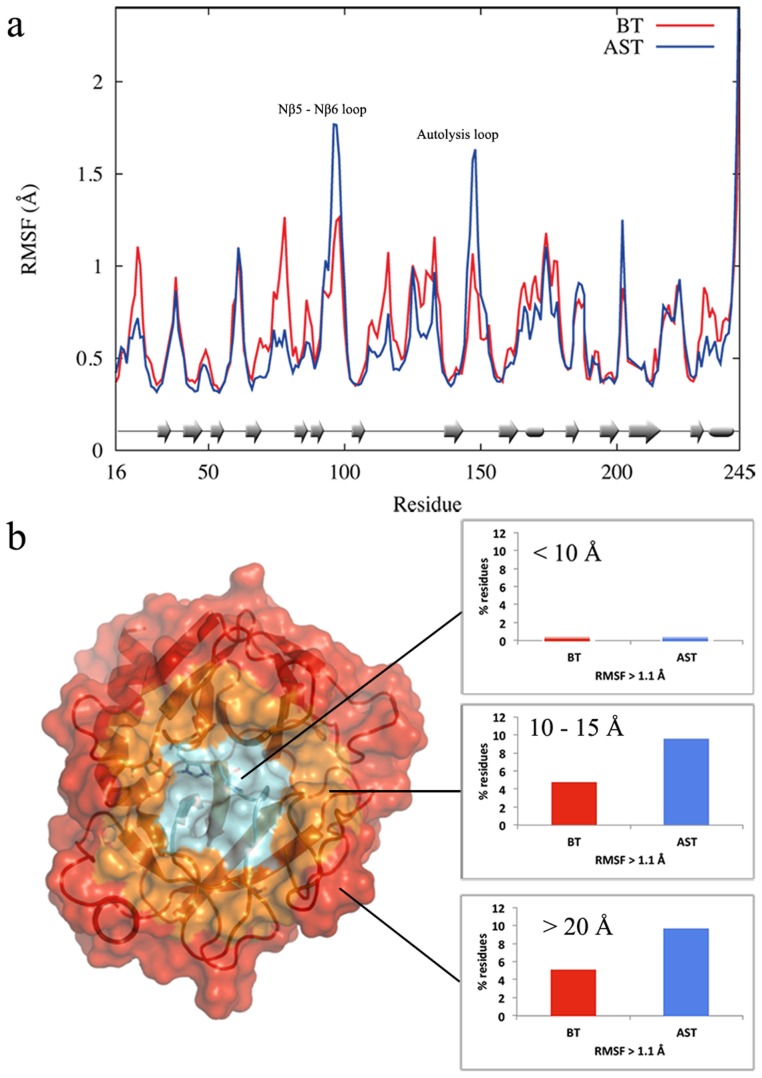
Residual fluctuations and structural location of mobile residues. (a) Average protein backbone fluctuations for bovine (BT) and salmon (AST) trypsin during 100 ns of MD in the reactant state. (b) The percentage of residues with RMSFs>1.1 Å, in spherical regions centered on the active site, indicate that both enzymes have a hard core and a softer outer region, in which AST has a higher prevalence of mobile residues.

**Figure 3:**
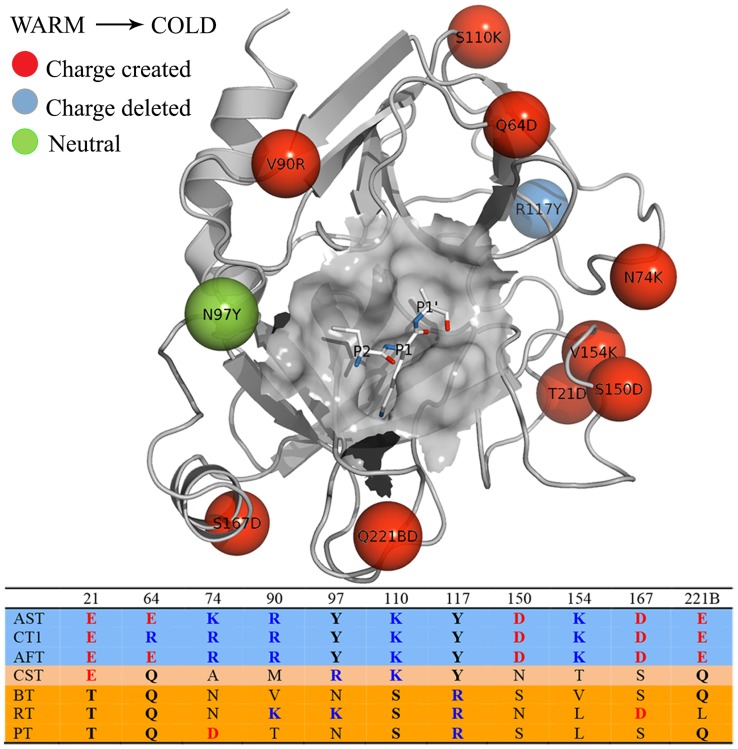
Important mutations between bovine and salmon trypsin. Key mutations between bovine and salmon trypsin and their location in the 3D structure, identified based on sequence alignments (inset below) of several warm-active vs cold-active trypsins. A red sphere indicates that a neutral residue in bovine trypsin is mutated to a charged one in salmon trypsin, whereas a blue sphere denotes the reverse type of substitution, and a green sphere denotes a neutral residue in both trypsins. Abbreviations used: AST - anionic salmon trypsin, AFT - Antarctic fish trypsin, CT1 - Atlantic cod trypsin, CST - cationic salmon trypsin, RT - rat trypsin, PT - Pig trypsin and BT - bovine trypsin.

To further examine the flexibility hypothesis, we carried out additional 100 ns simulations at both the transition and reactant states for BT and AST. As in Refs. [10] and [24], we again find that the mobility of the active site is low and practically identical in the two enzymes (Fig. S2). Furthermore, the overall protein backbone RMSFs are very similar with calculated values of 0.65 Å and 0.66 Å for BT in the reactant and transition state, respectively, while the corresponding values for AST are 0.61 Å and 0.65 Å. A plot of the average backbone positional fluctuations versus amino acid sequence (Fig. 2a), however, shows as expected that there are local differences in mobility and that these mainly are found on the protein surface. For example, Tyr97 and Asp150 in AST are significantly more flexible than their corresponding BT residues. Both Tyr97, situated in the Nβ5-Nβ6 loop, and Asp150 of the so-called autolysis loop are also conserved through different cold-adapted trypsins (Fig. 3). Moreover, further analysis of the backbone RMSFs shows that the prevalence of residues with high mobility, measured radially from the active site, differs significantly between bovine and salmon trypsin (Fig. 2b). That is, while both enzymes become more flexible further away from the active site, the cold-adapted protein has a markedly higher prevalence of residues with high RMSF values beyond 10 Å from the active site. The conclusion is thus that both enzymes have a relatively rigid core and softer outer regions, but that the surface regions of the cold-adapted enzyme are, at least locally, softer than for the warm-adapted protein.

### Point-mutations can change the thermodynamic activation parameters

Both the energetic and mobility analysis above strongly suggest that the surface of the cold-adapted enzyme is softer compared to its warm-active counterpart and the key question now is how this could be accomplished. Here, ultra-high resolution (0.75–1.0 Å) crystal structures of both BT [25] and AST [26] turn out to provide valuable clues since a large number of surface bound water molecules are resolved in these structures. Such water molecules very often allow polar surface side-chains, like those of Asn, Gln, Ser and Thr, to engage in extensive H-bond networks back to the protein surface. Charged surface side-chains, on the other hand, preferentially point out into solution due to their stronger requirement for solvation by bulk water. Analysis of the key mutations identified in Fig. 3 with these high-resolution structures [25], [26] reveals some basic principles for how the protein surface can be made softer by point-mutations.

**Figure 4:**
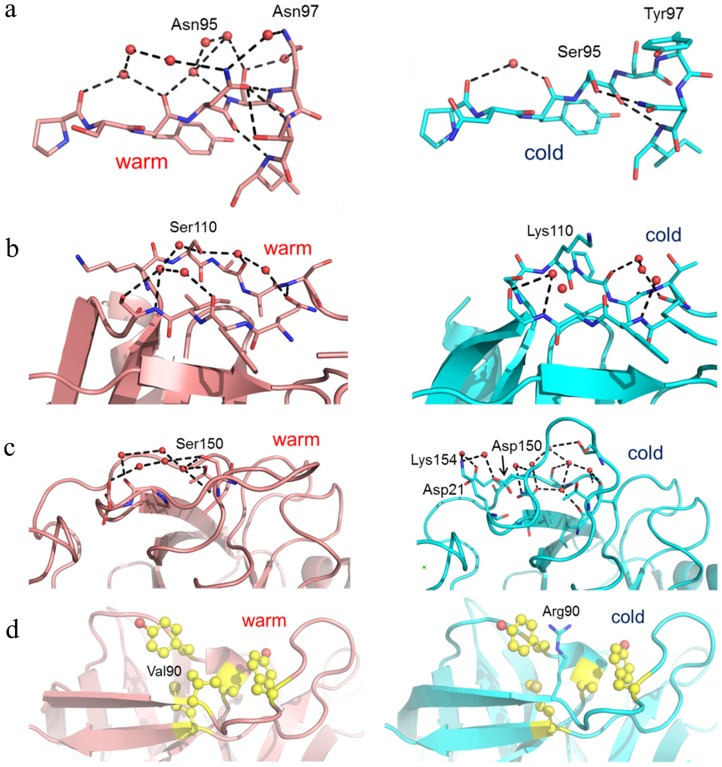
Mechanisms for increasing surface softness in salmon trypsin (cyan) relative to bovine (red) trypsin. (a) Disruption of an H-bond network by mutation of polar residue into a less polar (Asn97Tyr). (b) Disruption of an H-bond network by mutation of a polar residue into a charged one (Ser110Lys). (c) Complete change of H-bond network and loop orientation (Ser150Asp and correlated muations). (d) Destabilization of the packing of hydrophobic surface patches by mutation of a nonpolar residue into a charged one (Val 90Arg).

First, the surface can be softened by disrupting a water mediated H-bond network through mutation of a polar to a less polar residue. This is exemplified in the trypsins by mutation of Asn97 in BT to Tyr97 in AST, which largely abolishes such a network (Fig. 4a). Second, a surface H-bonding network can also be disrupted by mutation of a polar residue into a charged one, since the latter may prefer to interact with bulk solvent. This is exemplified by the mutation of Ser110 in BT to Lys110 in AST (Fig. 4b). A single or few mutations may also completely change one surface H-bond network into another such as the structurally correlated Thr21Glu, Ser150Asp and Val154Lys mutations, which drastically affect the conformation of the autolysis loop and make the active site region more solvent accessible in AST (Fig. 4c). Finally, one can also identify mutations that destabilize the packing of hydrophobic surface patches by mutation of a nonpolar residue into a charged one. This is, e.g., the case with the mutation of Val90 in BT to Arg90 in AST (Fig. 4d).

Of the mutations mentioned above, we will focus on Asn97Tyr and Ser150Asp since both of these are strictly conserved in the cold-adapted trypsins (Fig. 3) and are also the ones showing the largest increase in flexibility compared to the warm-adapted enzyme (Fig. 2a). We thus again calculated free energy profiles at different temperatures to obtain Arrhenius plots for the four cross-species mutations BT_N97Y_, BT_S150D_, AST_Y97N_ and AST_D150S_, in order to be able to predict their thermodynamic activation parameters. The resulting calculated free activation energies remain essentially unchanged by the mutations (Table 1), which underscores the general notion that mutations far away from the active site do not significantly affect catalytic rates [27]. However, what is remarkable is that the calculations predict significant changes in ΔH^‡^ and ΔS^‡^ for most mutations, but that these are again nearly perfectly compensating. Thus, both the BT_N97Y_ and BT_S150D_ mutations markedly lower the activation enthalpy and make the entropy more negative compared to the wild-type bovine enzyme and they become more like the cold-adapted AST. For the reverse mutations, AST_D150S_ renders the cold-adapted enzyme more mesophilic-like with a significantly raised ΔH^‡^ and a more positive ΔS^‡^. This is also seen by the predicted effect the autolysis loop structure, which approaches the bovine conformation (Fig. S3). The AST_Y97N_ mutation, on the other hand, yields relatively smaller effects on both the activation parameters and 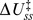. This probably just reflects the fact that correlated mutations (e.g., AST_S95N_) may be needed to build up the native bovine H-bond network involving the Nβ5-Nβ6 loop (Fig. 4a), so that a single point-mutation does not suffice.

**Figure 5:**
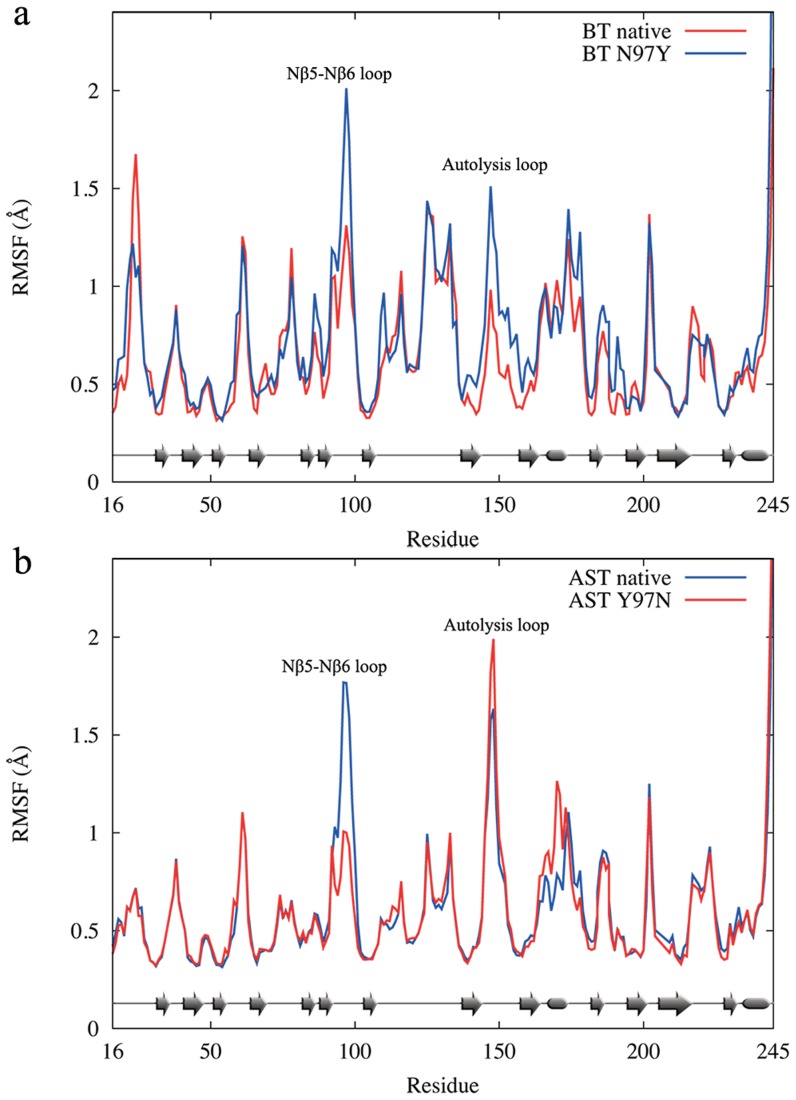
Comparison of residual fluctuations for native and mutant trypsins. (**a**) Average backbone RMSF profiles in the reactant state for native bovine trypsin and the N97Y mutant and (**b**) for native salmon trypsin and theY97N mutant.

Since both of the residues mutated are involved in distinct H-bond networks in the bovine enzyme, which appear to rigidify the surface, it is logical that a single mutation could destroy such a network and make the surface softer. In this respect, it would seem more difficult to conversely rigidify the surface by a single mutation, as in the case of AST_Y97N_, if that requires the creation of a new H-bond network. It is also noteworthy here, that the single BT_N97Y_ mutation is predicted to yield values of ΔH^‡^, TΔS^‡^ and 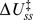 that are almost identical to those of native AST and *k_cat_* is predicted to increase 19-fold at 4°C for this mutation. That mutation of residue 97, either from BT to AST or vice versa, has a pronounced effect on the backbone mobility of the Nβ5-Nβ6 loop is also evident (Fig. 5), where Asn consistently reduces positional fluctuations whereas Tyr increases them.

## Discussion

In this work, we have addressed the problem of the structural origin of what appears to be a universal characteristic feature of cold-adapted enzymes, namely a reduced enthalpy and more negative entropy of activation. This was done using Atlantic salmon and bovine trypsin, cold- and warm-active, respectively, as models for the phenomenon. With very extensive all-atom computer simulations, using the EVB method to describe the catalytic reaction, reliable Arrhenius plots for the temperature dependence of the activation free energy could be obtained. It should be pointed out here that there is probably no other feasible way at present to calculate Arrhenius plot from first principles. It is rather remarkable that these simulations clearly reproduce the characteristic balance between activation enthalpy and entropy for cold-adapted versus warm-active enzymes, as well as the higher catalytic rate of AST compared to BT. Nevertheless, the activation free energies are similar at room temperature, thus reflecting a near perfect compensation between the former activation parameters.

The origin of the catalytically beneficial low activation enthalpy at low temperatures (accompanied by a more negative entropy) is found to be associated not with the active site but with the protein surface. From an evolutionary point of view this is perhaps not so surprising since any mutations in or near an optimized active site are likely to cause drastic rate reductions. What is rather surprising, however, is that it is the softness of the protein-water surface that appears to regulate the activation enthalpy-entropy balance. The simple picture that emerges is thus that the enzymes have a relatively rigid core, where the probability of successful adaptive mutations is low, surrounded by a softer outer matrix (Fig. 2b) whose properties can be fine-tuned by evolution.

While our earlier study of citrate synthases [10] also identified the same potential energy terms as responsible for the altered activation enthalpy-entropy balance, the structural origin of the effect remained obscure. Here, with the help of ultra-high resolution crystal structures, the actual structural “mechanisms” by which the surface softness is altered could finally be elucidated. Moreover, computational predictions of the effects of surface mutations were made that strongly support these conclusions. In particular, we identified extensive H-bond networks between polar surface groups and bound water molecules in the mesophilic enzyme that rigidify the surface, and several cold-adaptive mutations soften it by disrupting these networks. However, there are also examples of mutations in the cold-adapted enzyme (e.g., Val90Arg and Val154Lys) that appear to destabilize local hydrophobic surface patches. In view of the above findings, it is understandable that structural bioinformatics analysis has not yielded any consistent common descriptors of cold-adapted enzymes [5]. That is, since we identified several different types of mechanisms for surface destabilization and since the effects are often very local it is unlikely that there are distinct global descriptors that can capture them.

It is also noteworthy that the computer simulations predict that the enzyme ΔH^‡^ and ΔS^‡^ activation parameters can change significantly due to a single surface mutation. Such phenomena have, in fact, also been experimentally observed for other orthologous enzymes [28]. At first this may seem as a mysterious long-range effect on catalysis, but it should be emphasized that the activation free energies, and hence the catalytic rates, remain essentially unchanged. Instead it is the surface properties that are altered by such point mutations and mutations that soften the surface can apparently reduce the activation enthalpy of the catalyzed reaction at the expense of a more negative activation entropy. Such surface alterations are, however, beneficial for adaptation to low temperatures as they simultaneously make the rate more temperature insensitive and presumably also counteract the structural rigidity imposed by the reduction in temperature.

It is, of course, important to also try to address the generality of the present findings. In this respect, it should first be recalled that the characteristic trends with regard to activation enthalpy and entropy for cold-adapted enzymes appear to be completely universal, without known exceptions [5]. Two different types of enzymes (citrate synthases [10] and trypsins) have now been analyzed by extensive reaction simulations, which reproduce the observed behavior of warm- and cold-adapted orthologs, and which identify protein surface softness as the key variable. There is also other circumstantial evidence pointing towards surface properties, and flexibility in particular [29], [30]. Furthermore, the active site residues are basically always conserved between highly similar orthologous warm- and cold-adapted enzymes, which makes the idea that active site fluctuations would be substantially different very unlikely. It does therefore not seem far-fetched to assume that the difference in protein surface properties found here is likely to be a general feature of cold-adapted enzymes. It is further noteworthy that significantly altered kinetics and thermostability due to amino acid changes at a few sites distant from the active site have also been observed in dehydrogenases that are rate-limited by conformational changes rather than by chemistry [29], [30], as in the case of trypsin [19].

While the effects discussed herein pertain to the catalytic rates of the enzymes, their influence on thermostability is more difficult to assess. As mentioned, reduced thermostability is also an apparent universal characteristic of enzymes adapted to cold environments The net stability of folded proteins normally on the order of 10 kcal/mol, and is the result of large compensatory contributions. This, of course, makes it difficult to point out a single factor to explain differences in thermostability. However, previous studies of trypsin [31] indicate that the stability of a few loops and the C-terminal helix are important factors when explaining the difference in thermostability of cold- and warm-adapted trypsins. While our results also identify the same loop regions as important for adaptation to cold, experimental characterization is needed to examine whether these amino acid substitutions only change the catalytic rate or whether they affect thermostability as well.

## Supporting Information

Figure S1 **A solvation sphere with 35 Å radius covering the entire enzyme was used in all simulations.**
(TIF)

Figure S2 **Active site residue RMSFs from MD simulations for bovine and anionic salmon trypsin.** All RMSF values were calculated based on 100 ns MD simulations of the reactant (RS) and transition state (TS). The three rightmost residues denote those of the tripeptide substrate.
(TIF)

Figure S3 **MD simulations predict that the single-point mutation D150S in the autolysis loop in anionic salmon trypsin (AST) makes the orientation approach the bovine conformation.** Calculated thermodynamic activation parameters for the ASTD150S mutant also render the cold-adapted enzyme more mesophilic-like.
(TIF)

Text S1 **Supplementary methods.** Additional information on the calibration of the EVB reaction surface and details of the simulations are described.
(DOCX)

## References

[1] Feller G, Gerday C (2003) Psychrophilic enzymes: hot topics in cold adaptation. Nat Rev Microbiol 1: 200–208. 15035024 10.1038/nrmicro773

[2] Low PS, Bada JL, Somero GN (1973) Temperature adaptation of enzymes: roles of the free energy, the enthalpy, and the entropy of activation. Proc Natl Acad Sci U S A 70: 430–432. 4510286 10.1073/pnas.70.2.430 PMC433275

[3] Johnston IA, Walesby NJ, Davison W, Goldspink G (1975) Temperature adaptation in myosin of Antarctic fish. Nature 254: 74–75. 123042 10.1038/254074a0

[4] Privalov PL, Tiktopulo EI, Tischenko VM (1979) Stability and mobility of the collagen structure. J Mol Biol 127: 203–216. 430563 10.1016/0022-2836(79)90240-7

[5] Siddiqui KS, Cavicchioli R (2006) Cold-adapted enzymes. Annu Rev Biochem 75: 403–433. 16756497 10.1146/annurev.biochem.75.103004.142723

[6] Fields PA, Somero GN (1998) Hot spots in cold adaptation: localized increases in conformational flexibility in lactate dehydrogenase A4 orthologs of Antarctic notothenioid fishes. Proc Natl Acad Sci U S A 95: 11476–11481. 9736762 10.1073/pnas.95.19.11476 PMC21668

[7] Somero GN (1995) Proteins and temperature. Annu Rev Physiol 57: 43–68. 7778874 10.1146/annurev.ph.57.030195.000355

[8] Lonhienne T, Gerday C, Feller G (2000) Psychrophilic enzymes: revisiting the thermodynamic parameters of activation may explain local flexibility. Biochim Biophys Acta 1543: 1–10. 11087936 10.1016/s0167-4838(00)00210-7

[9] Smalås AO, Heimstad ES, Hordvik A, Willassen NP, Male R (1994) Cold adaption of enzymes: structural comparison between salmon and bovine trypsins. Proteins 20: 149–166. 7846025 10.1002/prot.340200205

[10] Bjelic S, Brandsdal BO, Åqvist J (2008) Cold adaptation of enzyme reaction rates. Biochemistry 47: 10049–10057. 18759500 10.1021/bi801177k

[11] Warshel A. (1991) Computer Modeling of Chemical Reactions in Enzymes and Solutions. New York: John Whiley & Sons.

[12] Åqvist J, Warshel A (1993) Simulation of enzyme reactions using valence bond force fields and other hybrid quantum/classical approaches. Chem Rev 93: 2523–2544.

[13] Helland R, Leiros I, Berglund GI, Willassen NP, Smalås AO (1998) The crystal structure of anionic salmon trypsin in complex with bovine pancreatic trypsin inhibitor. Eur J Biochem 256: 317–324. 9760170 10.1046/j.1432-1327.1998.2560317.x

[14] Helland R, Otlewski J, Sundheim O, Dadlez M, Smalås AO (1999) The crystal structures of the complexes between bovine beta-trypsin and ten P1 variants of BPTI. J Mol Biol 287: 923–942. 10222201 10.1006/jmbi.1999.2654

[15] Marelius J, Kolmodin K, Feierberg I, Åqvist J (1998) Q: a molecular dynamics program for free energy calculations and empirical valence bond simulations in biomolecular systems. J Mol Graph Model 16: 213–225, 261. 10522241 10.1016/s1093-3263(98)80006-5

[16] Jorgensen WL, Maxwell DS, Tirado-Rives J (1996) Development and testing of the OPLS all-atom force field on conformational energetics and properties of organic liquids. J Am Chem Soc 118: 11225–11236.

[17] Kaminski GA, Friesner RA, Tirado-Rives J, Jorgensen WL (2001) Evaluation and reparametrization of the OPLS-AA force field for proteins via comparison with accurate quantum chemical calculations on peptides. J Phys Chem B 105: 6474–6487.

[18] Štrajbl M, Florián J, Warshel A (2000) Ab initio evaluation of the potential surface for general base- catalyzed methanolysis of formamide: A reference solution reaction for studies of serine proteases. J Am Chem Soc 122: 5354–5366.

[19] Fersht A (1999) Structure and mechanism in protein science: A guide to enzyme catalysis and protein folding. New York: W. H. Freeman and Company.

[20] Kraut J (1977) Serine proteases - structure and mechanism of catalysis. Annu Rev Biochem 46: 331–358. 332063 10.1146/annurev.bi.46.070177.001555

[21] Warshel A, Narayszabo G, Sussman F, Hwang JK (1989) How do serine proteases really work. Biochemistry 28: 3629–3637. 2665806 10.1021/bi00435a001

[22] Outzen H, Berglund GI, Smalås AO, Willassen NP (1996) Temperature and pH sensitivity of trypsins from Atlantic salmon (Salmo salar) in comparison with bovine and porcine trypsin. Comp Biochem Physiol B Biochem Mol Biol 115: 33–45. 8896331 10.1016/0305-0491(96)00081-8

[23] Radzicka A, Wolfenden R (1995) Transition-state and multisubstrate analog inhibitors. Method Enzymol 249: 284–312. 10.1016/0076-6879(95)49039-6 7791615

[24] Brandsdal BO, Heimstad ES, Sylte I, Smalås AO (1999) Comparative molecular dynamics of mesophilic and psychrophilic protein homologues studied by 1.2 ns simulations. J Biomol Struct Dyn 17: 493–506. 10636084 10.1080/07391102.1999.10508380

[25] Liebschner D, Dauter M, Brzuszkiewicz A, Dauter Z (2013) On the reproducibility of protein crystal structures: five atomic resolution structures of trypsin. Acta Crystallogr D 69: 1447–1462. 23897468 10.1107/S0907444913009050 PMC3727327

[26] Leiros HKS, McSweeney SM, Smalås AO (2001) Atomic resolution structures of trypsin provide insight into structural radiation damage. Acta Crystallogr D 57: 488–497. 11264577 10.1107/s0907444901000646

[27] Morley KL, Kazlauskas RJ (2005) Improving enzyme properties: when are closer mutations better? Trends Biotechnol 23: 231–237. 15866000 10.1016/j.tibtech.2005.03.005

[28] Ghanem M, Li L, Wing C, Schramm VL (2008) Altered thermodynamics from remote mutations altering human toward bovine purine nucleoside phosphorylase. Biochemistry 47: 2559–2564. 18281956 10.1021/bi702132e

[29] Johns GC, Somero GN (2004) Evolutionary convergence in adaptation of proteins to temperature: A4-lactate dehydrogenases of Pacific damselfishes (Chromis spp.). Mol Biol Evol 21: 314–320. 14660697 10.1093/molbev/msh021

[30] Holland LZ, McFall-Ngai M, Somero HN (1997) Evolution of lactate dehydrogenase-A homologs of barracuda fishes (genus Sphyraena) from different thermal environments: differences in kinetic properties and thermal stability are due to amino acid substitutions outside the active site. Biochemistry 36: 3207–3215. 9115998 10.1021/bi962664k

[31] Leiros HKS, Willassen NP, Smalås AO (1999) Residue determinants and sequence analysis of cold-adapted trypsins. Extremophiles 3: 205–219. 10484177 10.1007/s007920050118

