# Supplementary figures and images for: Protein Surface Softness Is the Origin of Enzyme Cold-Adaptation of Trypsin
Source: PLoS Comput Biol. 2014 Aug 28;10(8):e1003813. doi: 10.1371/journal.pcbi.1003813 (PMC4148182; doi:10.1371/journal.pcbi.1003813)

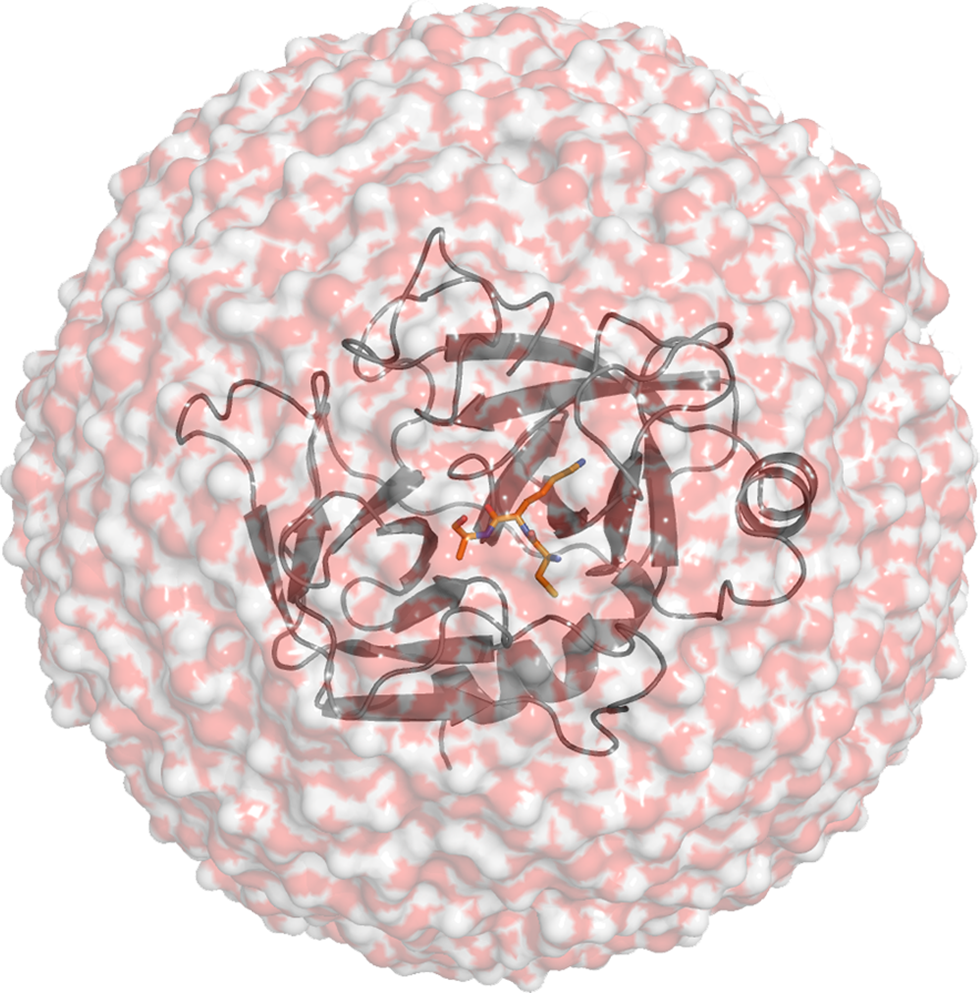

Supplement: Figure S1 — A solvation sphere with 35 Å radius covering the entire enzyme was used in all simulations. (TIF) [file pcbi.1003813.s001.tif]

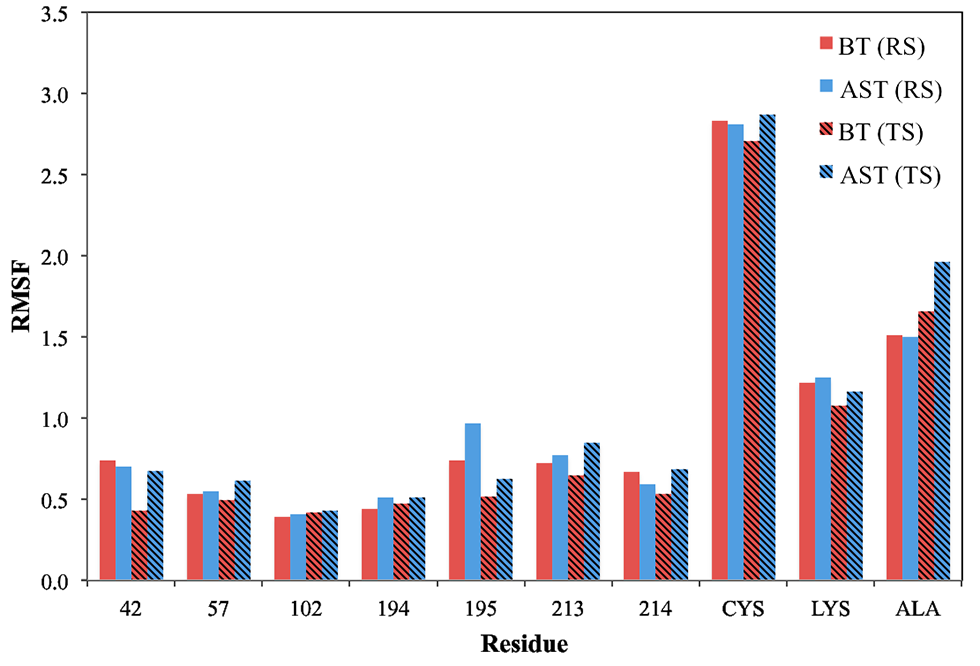

Supplement: Figure S2 — Active site residue RMSFs from MD simulations for bovine and anionic salmon trypsin. All RMSF values were calculated based on 100 ns MD simulations of the reactant (RS) and transition state (TS). The three rightmost residues denote those of the tripeptide substrate. (TIF) [file pcbi.1003813.s002.tif]

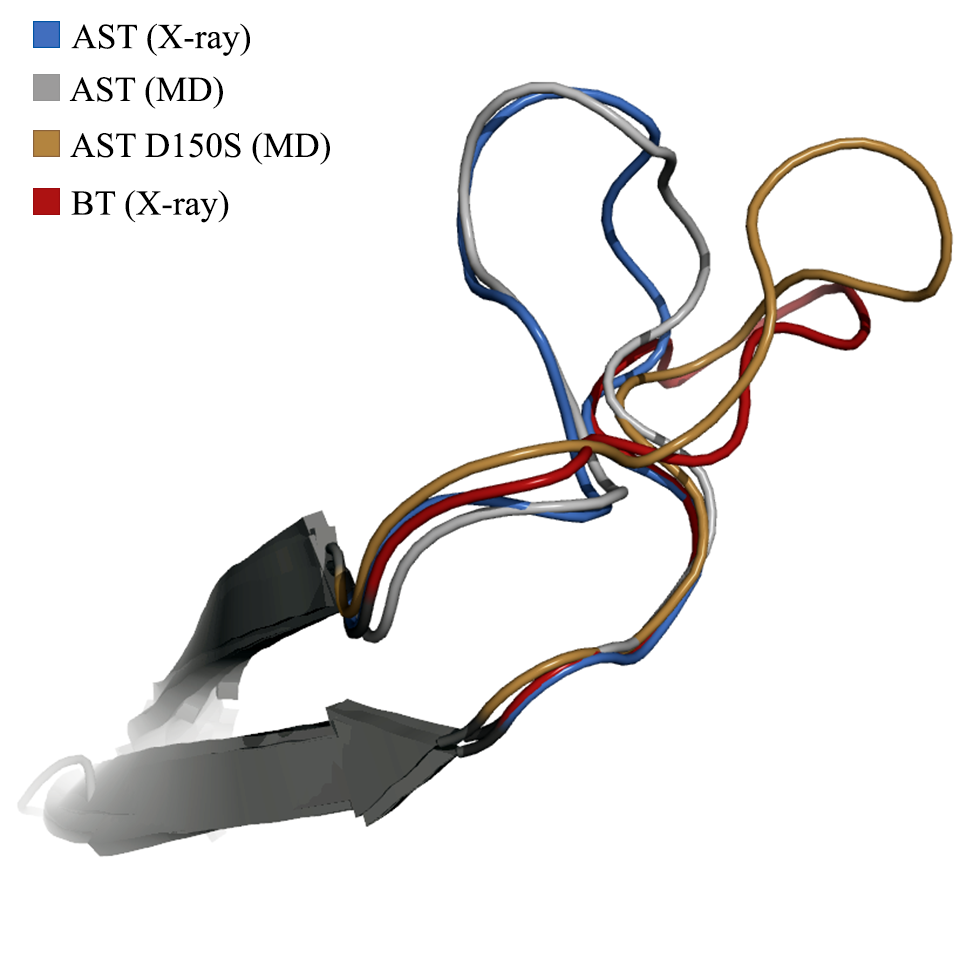

Supplement: Figure S3 — MD simulations predict that the single-point mutation D150S in the autolysis loop in anionic salmon trypsin (AST) makes the orientation approach the bovine conformation. Calculated thermodynamic activation parameters for the ASTD150S mutant also render the cold-adapted enzyme more mesophilic-like. (TIF) [file pcbi.1003813.s003.tif]
