# Supplementary material for: Protein Surface Softness Is the Origin of Enzyme Cold-Adaptation of Trypsin
Source: PLoS Comput Biol. 2014 Aug 28;10(8):e1003813. doi: 10.1371/journal.pcbi.1003813 (PMC4148182; doi:10.1371/journal.pcbi.1003813)
Supplement: Text S1 — Supplementary methods. Additional information on the calibration of the EVB reaction surface and details of the simulations are described. (DOCX) [file pcbi.1003813.s004.docx]

# Supplementary Methods

**EVB reaction surface**

Formation of the tetrahedral intermediate in the acylation reaction of the psychrophilic and mesophilic trypsins was described with the EVB method [1,2] as a concerted reaction between two valence bond structures (Fig. 1). The EVB potential energy surface was calibrated using the imidazole catalyzed methanolysis of formamide in water [3] as a reference reaction. The energetics of this reference reaction in water was fitted to available *ab initio* data (including solvation by the Langevin dipole model) from ref. [3] by adjusting the off-diagonal *H_12_* term of the EVB Hamiltonian, as well as the gas-phase energy difference ${\Delta\alpha}$ between the two VB states [1,2]. The target values for activation and reaction free energy of the reference reaction in a solvent cage corresponding to a nucleophile concentration of 55M were 26 and 20 kcal/mol, respectively. The MD/FEP umbrella sampling simulations reproduce these free energies with the relevant EVB parameters adjusted to ${\Delta\alpha}$ = -213 kcal/mol and *H*_12_ = 111 kcal/mol.

**Simulations details**

Models of psycrophilic and mesophilic trypsin were constructed from the crystallographic structures with PDB codes 1BZX [4] and 3BTK [5]. As a model for the substrate, the P2, P1 and P1’ residues (Cys-Lys-Ala corresponding to residues 514 – 516) of the trypsin complex with bovine pancreatic trypsin inhibitor (BPTI) were used. Missing parameters and charges were assigned using Impact 5.6 (Schrödinger, LLC, New York, NY, 2011). The proteins were immersed into a spherical droplet of TIP3P water molecules [6] with a radius of 35 Å centered on the scissile bond of the substrate. Long-range electrostatics was treated using a multipole expansion method [7] and a direct 10 Å nonbonded cutoff for solvent-solvent and solute-solvent interactions. No cutoffs were applied to interactions involving the reacting fragments. All systems were heated from 1 K to the final temperature during 31 ps, using a stepwise scheme, followed by an equilibration period of 100 ps. The SHAKE algorithm [8] was used to constrain bonds and angles on solvent molecules. A time step of 1 fs was used for the production phase, and the temperature was controlled by coupling to an external bath. The reacting region of the EVB model consisted of all atoms from C_β_ in the side chain of His57 and Ser195, the Lys515 C_α_-atom and all atoms in the amide bond between Lys515 and Ala516 (C, O, N and H).

**References:**

1. Warshel A (1991) Computed Modeling of Chemical Reactions in Enzymes and Solutions. New York: John Whiley & Sons.

2. Åqvist J, Warshel A (1993) Simulation of enzyme reactions using valence bond force fields and other hybrid quantum/classical approaches. Chem Rev 93: 2523-2544.

3. Štrajbl M, Florián J, Warshel A (2000) Ab initio evaluation of the potential surface for general base-catalyzed methanolysis of formamide: A reference solution reaction for studies of serine proteases. J Am Chem Soc 122: 5354-5366.

4. Helland R, Leiros I, Berglund GI, Willassen NP, Smalås AO (1998) The crystal structure of anionic salmon trypsin in complex with bovine pancreatic trypsin inhibitor. Eur J Biochem 256: 317-324.

5. Helland R, Otlewski J, Sundheim O, Dadlez M, Smalås AO (1999) The crystal structures of the complexes between bovine beta-trypsin and ten P1 variants of BPTI. J Mol Biol 287: 923-942.

6. Jorgensen WL, Chandrasekhar J, Madura JD, Impey RW, Klein ML (1983) Comparison of simple potential functions for simulating liquid water. J Chem Phys 79: 926-935.

7. Lee FS, Warshel,A (1992) A local reaction field method for fast evaluation of long-range electrostatic interactions in molecular simulations. J Chem Phys 97: 3100-3107.

8. Ryckaert JP, Ciccotti G, Berendsen HJC (1977) Numerical-integration of cartesian equations of motion of a system with constraints - molecular-dynamics of N-alkanes. J Comput Phys 23: 327-341.
